# Simulation of Daily Snapshot Rhythm Monitoring to Identify Atrial Fibrillation in Continuously Monitored Patients with Stroke Risk Factors

**DOI:** 10.1371/journal.pone.0148914

**Published:** 2016-02-16

**Authors:** Yuichiro Yano, Philip Greenland, Donald M. Lloyd-Jones, Emile G. Daoud, Jodi L. Koehler, Paul D. Ziegler

**Affiliations:** 1 Department of Preventive Medicine, Northwestern University Feinberg School of Medicine, Chicago, Illinois, United States of America; 2 Department of Medicine, Division of Cardiology, Richard M. Ross Heart Hospital, The Ohio State University Medical Center, Columbus, Ohio, United States of America; 3 Medtronic Inc., Minneapolis, Minnesota, United States of America; Indiana University, UNITED STATES

## Abstract

**Background:**

New technologies are diffusing into medical practice swiftly. Hand-held devices such as smartphones can record short-duration (e.g., 1-minute) ECGs, but their effectiveness in identifying patients with paroxysmal atrial fibrillation (AF) is unknown.

**Methods:**

We used data from the TRENDS study, which included 370 patients (mean age 71 years, 71% men, CHADS_2_ score≥1 point: mean 2.3 points) who had no documentation of atrial tachycardia (AT)/AF or antiarrhythmic or anticoagulant drug use at baseline. All were subsequently newly diagnosed with AT/AF by a cardiac implantable electronic device (CIED) over one year of follow-up. Using a computer simulation approach (5,000 repetitions), we estimated the detection rate for paroxysmal AT/AF via daily snapshot ECG monitoring over various periods, with the probability of detection equal to the percent AT/AF burden on each day.

**Results:**

The estimated AT/AF detection rates with snapshot monitoring periods of 14, 28, 56, 112, and 365 days were 10%, 15%, 21%, 28%, and 50% respectively. The detection rate over 365 days of monitoring was higher in those with CHADS_2_ scores ≥2 than in those with CHADS_2_ scores of 1 (53% vs. 38%), and was higher in those with AT/AF burden ≥0.044 hours/day compared to those with AT/AF burden <0.044 hours/day (91% vs. 14%; both *P*<0.05).

**Conclusions:**

Daily snapshot ECG monitoring over 365 days detects half of patients who developed AT/AF as detected by CIED, and shorter intervals of monitoring detected fewer AT/AF patients. The detection rate was associated with individual CHADS_2_ score and AT/AF burden.

**Trial Registration:**

ClinicalTrials.gov NCT00279981

## Introduction

One-third of atrial fibrillation (AF) patients have no obvious symptoms, and the prevalence of paroxysmal AF is 25–60% of total AF cases [1–5]. Asymptomatic and paroxysmal AF is associated with a two- to three-fold higher risk of stroke than the absence of AF [5–7], so the challenge is to identify those with paroxysmal AF prior to the occurrence of stroke events and appropriate use of anticoagulation therapy in AF patients [8,9].

National AF guidelines from the United States and Europe recommend AF screening for patients with stroke risk factors [10,11]. The effort has been hampered, however, by the lack of effective screening approaches in terms of accuracy and sensitivity, cost-effectiveness, and patient acceptability/convenience. Newly available technology, such as smartphone based single-lead electrocardiography (ECG) [12,13] and hand-held ECG [14], were shown to record AF accurately by comparing it with a contemporaneous 12-lead ECG. These devices are relatively inexpensive, accessible, and easy to use in many developed countries, so patients can readily use them over long periods of time [15]. Since paroxysmal AF may be unassociated with symptoms and occurs sporadically, it remains uncertain to what extent short time periods of smartphone ECG monitoring can capture paroxysmal AF events in ambulatory outpatients, even when used frequently over a long period (e.g., daily snapshot monitoring over 365 days). The TRENDS study provides a unique opportunity to elucidate this issue, since some patients were enrolled who had no documentation of AF at baseline and then were subsequently diagnosed with atrial tachycardia (AT)/AF by a cardiac implantable electronic device (CIED) over one year of follow-up [7,16,17].

Using a computer simulation approach in the TRENDS study, we sought to estimate the sensitivity of repeated daily snapshot ECG monitoring over various durations of monitoring (14, 28, 56, 112, and 365 days) in detecting AT/AF.

## Material and Methods

### Study Population

The present analyses were based on a subset of patients enrolled in the TRENDS study. The design [18] and primary results [7] of the original TRENDS study have been published previously. In brief, TRENDS was a multicenter prospective study that enrolled 2,813 patients who had recently received CIEDs (i.e., pacemakers, implantable cardioverter defibrillators, or cardiac resynchronization therapy devices) for a class I-II indication. Participants in this analysis had a CHADS_2_ score ≥1 (including history of congestive heart failure or hypertension, age ≥75 years, diabetes mellitus, or prior stroke/transient ischemic attack) [9] and no history of AF at baseline but then newly detected AT/AF as confirmed by interrogation of the CIED during the first year post implant. Anticoagulants and antiarrhythmic drugs were prescribed at the discretion of the patient's managing physician. The study protocol was approved by the institutional review board of each participating center, and all patients provided written informed consent to participate in the TRENDS study.

### Device Programming

The patients were followed at 3-month intervals, at which time CIED diagnostic information was collected. Devices (Medtronic, Minneapolis, MN) were programmed to dual-chamber operation with active mode switching. AT/AF detection was programmed to the nominal settings (atrial rate >175 bpm lasting ≥20 seconds) to ensure consistent device programming. A prior study using similar detection algorithms showed >95% sensitivity and specificity for detection of AT/AF episodes and measurement of AT/AF burden [19].

Newly detected AT/AF was defined as device-detected AT/AF lasting >5 minutes on any day of the study. This duration threshold was selected because previous results have shown it to exclude most episodes of atrial oversensing that can lead to false-positive recordings of AF [20]. To obtain results with wide clinical applicability, we used a pragmatic approach by basing our analysis on the readily available diagnostic data within the device memory. We did not distinguish between episodes of AT, atrial flutter, or AF and did not examine the intracardiac electrograms, which were not available for every episode owing to device memory limitations.

### Analytic Plan

The purpose of the present analysis was to estimate the successful AT/AF detection rate (of any AT/AF greater than 5 minutes) via consecutive daily snapshot rhythm monitoring over various periods (14, 28, 56, 112, and 365 days). A Bernoulli distribution with the probability of success equal to the percent AT/AF burden on each day was used to determine if snapshot rhythm monitoring was successful for each day in the monitoring window. A Bernoulli distribution is a discrete distribution with two possible outcomes: success and failure. If any day during the monitoring window was deemed successful, AT/AF was considered detected by snapshot rhythm monitoring. To ensure a robust estimate of the number of patients with AT/AF detected by snapshot ECG monitoring, sampling was simulated 5000 times by our statistician (J.L.K.) and averaged to determine the “detection” rate.

We quantified the AT/AF burden, defined as the amount of time spent in AT/AF on a given day. For example, a patient who experienced 6 cumulative hours of AT/AF on a particular day would have an AF burden of 6 hours (or 25%) on that day. Average AT/AF burden per day over the monitoring period, maximum daily AT/AF burden, defined as the single day with maximal burden over the monitoring period, and the percentage of days over the monitoring period on which the patient experienced AT/AF were determined for each of the 370 patients.

We excluded the data of the initial 30 days after the device was implanted due to the possibility of transient AF caused by the implant procedure. Consequently, all monitoring windows began on day 31 following device implantation and extended for the corresponding evaluation period. AF needed only to be detected on a single day of the simulated monitoring window in order for the patient to be considered as being diagnosed with AF.

For the present study, we excluded 2,287 patients from the original TRENDS study population^9^ with a documented history of AF at baseline (n = 573), a CHADS_2_ score = 0 (n = 92), insufficient device trend data (n = 292), or no device-detected AF over the year of follow-up (n = 1,330). As a result, we included 526 patients with newly diagnosed AT/AF detected via CIED stored electrograms, of whom 156 were taking warfarin or antiarrhythmic drugs at baseline.

### Statistical Analysis

Continuous variables are presented as the mean ± SD and categorical variables are presented as percentages. Continuous variables were compared using a *t*-test and categorical variables using the chi-square test. Our analyses were performed with two approaches. In the primary analysis (n = 370), to exclude patients with potentially undocumented AF, we excluded 156 individuals who were taking warfarin or antiarrhythmic drugs at baseline. As a secondary objective (n = 526), we included and analyzed 156 patients taking warfarin or antiarrhythmic drugs at baseline, the assessment of which is more suitable for clinical practice. In the primary analyses, patients were classified based on CHADS_2_ score (intermediate risk [a CHADS_2_ score of 1] or high risk [a CHADS_2_ score of ≥2]) and AF burden (low AT/AF burden [less than the median average daily AT/AF burden of 0.044 hours/day] or high AT/AF burden [greater than or equal to the median average daily AT/AF burden of 0.044 hours/day]), and the rate for detecting AT/AF was calculated within each group. A significance level of *P*< 0.05 was used, and all statistical analyses were performed using SAS, version 9.2 (SAS Institute, Cary, North Carolina).

## Results

Table 1 shows a comparison of the baseline characteristics between patients who were included in the present primary analysis (n = 370) and those who were not included (n = 2,443). The included patients were more likely to be men, had a higher proportion of congestive heart failure, and a lower proportion of prior stroke/TIA than those not included. There were no differences in age, CHADS_2_ score, or blood pressure levels between the included and not included patients.

**Table 1:** The characteristics of patients who were included in the primary analysis, TRENDS study (n = 370).

Age, years	71±12
Men, %	71
Device type	
Pacemaker, %	43
ICD, %	32
CRT, %	25
CHADS_2_ score	2.3±1.0
0–1 point, %	25
2–6 point, %	75
Congestive heart failure, %	57
Hypertension, %	79
Age ≥75 years, %	43
Diabetes mellitus, %	30
Prior stroke/TIA, %	8
Coronary artery disease, %	64
Systolic BP, mmHg	134±23
Diastolic BP, mmHg	71±13
Drugs prescribed at baseline	
Warfarin, %	0
Aspirin, %	68
Class I/III antiarrhythmic drugs, %	0
Antiplatelet agent, %	25
Salicylates, %	5
NSAIDs, %	4
Antilipidemics, %	55

Data are expressed as means ± SD or percentage. ICD indicates implantable cardioverter-defibrillator; CRT, cardiac resynchronization therapy; TIA, transient ischemic attack; BP, blood pressure; NSAIDs, non-steroidal anti-inflammatory drugs.

**Fig 1:**
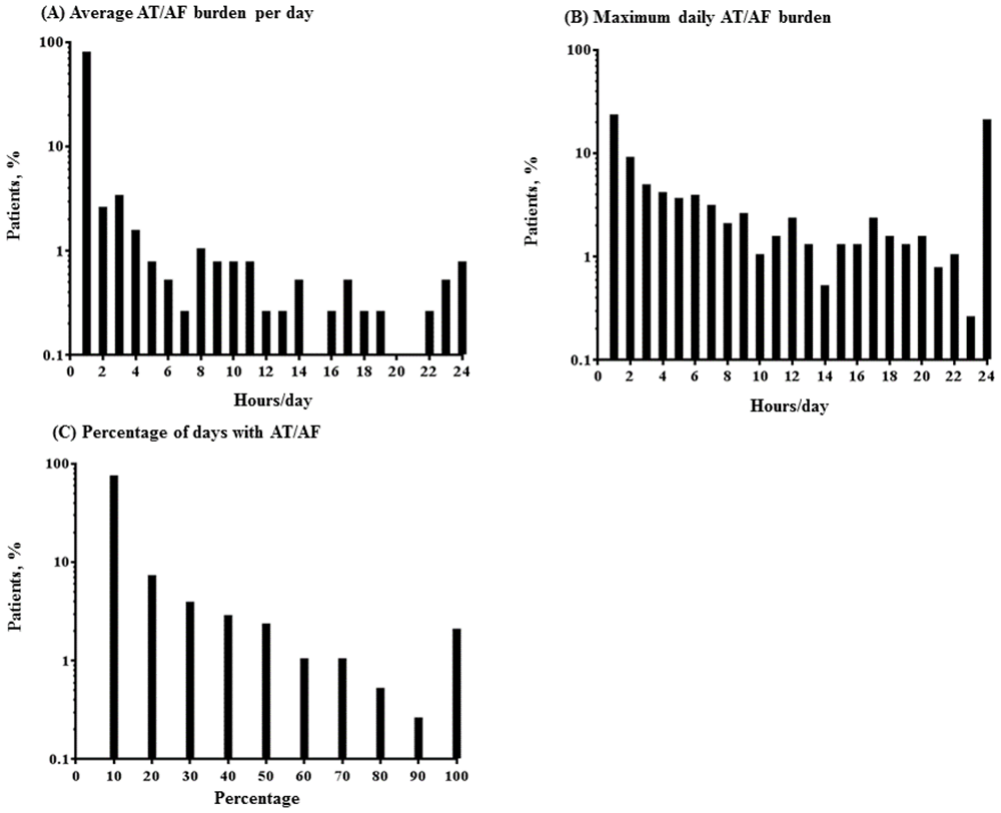
Histogram of the patient’s AT/AF burden (n = 370). AT/AF burden was defined as the amount of time spent in AT/AF on a given day. Average AT/AF burden per day over the monitoring period (A), maximum daily AT/AF burden, defined as the single day with maximal burden over the monitoring period (B), and the percentage of days over the monitoring period that the patient experienced AT/AF (C) were determined for each of the 370 patients in the primary analysis and are shown as a histogram. Each bar in Fig 1A and 1B indicates the proportion of individuals who were assigned into the corresponding group divided by 1-hour interval of the aggregated AT/AF events among 370 patients. For example, the bar of 6 hours/day in Fig 1A indicates that the proportion of patients with an average AT/AF burden between 5 to 6 hours/day among the 370 patients was 0.5%.

For the 370 participants, the median of average AT/AF burden, the maximum daily AT/AF burden, and the percentage of days with AT/AF were 0.044 hours/day (range = 0.0003–23.993), 5.5 hours/day (range = 0.1–24), and 1.6% (range = 0.3–100), respectively. Their distribution is shown in Fig 1. Approximately 80% were classified as having an average AT/AF burden over monitoring periods less than 1 hour/day, and as having an average percentage of days with AT/AF over monitoring periods less than 10%.

Table 2 shows the estimated detection rate of daily snapshot ECG monitoring over various periods (14, 28, 56, 112, and 365 days) among 370 patients with no prior history of AF and excluding those who were taking warfarin or antiarrhythmic drugs at baseline. At least 14 days of CIED data were available for all patients (n = 370), while the data over 365 days were available for 254 patients. Table 3 shows that snapshot monitoring over 14 days detected AT/AF in 37 of the 370 patients (10%). Prolongation of the snapshot monitoring up to 365 days increased the detection rate to 50%. Table 2 also shows that of the 92 patients whose CIEDs detected AT/AF during any of the 14 days of monitoring, snapshot monitoring detected AT/AF in 40% of them (n = 37). Of the 254 patients whose CIEDs detected AT/AF during any of the 365 days of monitoring, snapshot monitoring detected AT/AF in 50% of them (n = 126). The detection rate of snapshot monitoring for each monitoring window in 526 patients, including those who were taking warfarin or antiarrhythmic drugs at baseline, was slightly improved; e.g., the detection rate over 365 days of monitoring improved from 50% to 56%.

**Table 2:** Estimated detection rates for daily snapshot monitoring in patients with stroke risk factors.

Monitoring window of simulated daily snapshots	Number of patients with CIED data in window	Number of AT/AF patients detected by CIED in window (%)	Number of AT/AF patients detected by snapshot monitoring	
			% of total patients	% of patients with AT/AF in window
Primary analysis (n = 370)				
14 days	370	92 (24.9 [20.5–29.6]%)	37/370 (10.0 [7.1–13.5]%)	37/92 (40.2 [30.1–51.0]%)
28 days	366	122 (33.3 [28.5–38.4]%)	54/366 (14.8 [11.3–18.8]%)	54/122 (44.3 [35.3–53.5]%)
56 days	359	166 (46.2 [41.0–51.5]%)	77/359 (21.4 [17.3–26.1]%)	77/166 (46.4 [38.6–54.3]%)
112 days	351	229 (65.2 [60.0–70.2]%)	99/351 (28.2 [23.6–33.2]%)	99/229 (43.2 [36.7–49.9]%)
365 days	254	254 (100.0%)	126/254 (49.6 [43.3–55.9]%)	126/254 (49.6 [43.3–55.9]%)
Secondary analysis (n = 526)				
14 days	526	146 (27.8 [24.0–31.8]%)	77/526 (14.6 [11.7–18.0]%)	77/146 (52.7 [44.3–61.1]%)
28 days	521	189 (36.3 [32.1–40.6]%)	104/521 (20.0 [16.6–23.7]%)	104/189 (55.0 [47.6–62.3]%)
56 days	510	252 (49.4 [45.0–53.8]%)	135/510 (26.5 [22.7–30.5]%)	135/252 (53.6 [47.2–59.9]%)
112 days	496	327 (65.9 [61.6–70.1]%)	165/496 (33.3 [29.1–37.6]%)	165/327 (50.5 [44.9–56.0]%)
365 days	363	363 (100.0%)	203/363 (55.9 [50.6–61.1]%)	203/363 (55.9 [50.6–61.1]%)

Primary analysis was conducted for 370 patients, excluding those who were taking warfarin or antiarrhythmic drugs at baseline; secondary analysis was conducted for 526 patients, including those who were taking warfarin or antiarrhythmic drugs at baseline. Data are expressed as means [95% confidence intervals] of estimated AT/AF detection rates with the snapshot monitoring. CIED indicates cardiac implantable electronic device; AT, atrial tachycardia; AF, atrial fibrillation.

**Table 3:** Estimated detection rates for daily snapshot monitoring classified based on low/high CHADS_2_ score and low/high AT/AF burden (n = 370).

Monitoring window of simulated daily snapshots	Number of patients with CIED data in window	Number of AT/AF patients detected by CIED in window (%)	Number of AT/AF patients detected by snapshot monitoring	
			% of total patients	% of patients with AT/AF in window
CHADS_2_ score				
Intermediate risk group (n = 91)				
14 days	91	25 (27.5 [18.6–37.8]%)	11/91 (12.1 [6.2–20.6]%)	11/25 (44.0 [24.4–65.1]%)
28 days	91	37 (40.7 [30.5–51.5]%)	16/91 (17.6 [10.4–27.0]%)	16/37 (43.2 [27.1–60.5]%)
56 days	89	41 (46.1 [35.4–57.0]%)	20/89 (22.5 [14.3–32.6]%)	20/41 (48.8 [32.9–64.9]%)
112 days	88	59 (67.0 [56.2–76.7]%)	25/88 (28.4 [19.3–39.0]%)	25/59 (42.4 [29.6–55.9]%)
365 days	61	61 (100.0%)	23/61 (37.7 [25.6–51.0]%)	23/61 (37.7 [25.6–51.0]%)
High risk group (n = 279)				
14 days	279	67 (24.0 [19.1–29.5]%)	26/279 (9.3 [6.2–13.4]%)	26/67 (38.8 [27.1–51.5]%)
28 days	275	85 (30.9 [25.5–36.7]%)	38/275 (13.8 [10.0–18.5]%)	38/85 (44.7 [33.9–55.9]%)
56 days	270	125 (46.3 [40.2–52.4]%)	57/270 (21.1 [16.4–26.5]%)	57/125 (45.6 [36.7–54.7]%)
112 days	263	170 (64.6 [58.5–70.4]%)	74/263 (28.1 [22.8–34.0]%)	74/170 (43.5 [36.0–51.3]%)
365 days	193	193 (100.0%)	103/193 (53.4 [46.1–60.6]%)	103/193 (53.4 [46.1–60.6]%)
AT/AF burden				
Low burden Group (n = 185)				
14 days	185	22 (11.9 [7.6–17.4]%)	1/185 (0.5 [0.0–3.0]%)	1/22 (4.5 [0.1–22.8]%)
28 days	184	34 (18.5 [13.1–24.9]%)	2/184 (1.1 [0.1–3.9]%)	2/34 (5.9 [0.7–19.7]%)
56 days	183	57 (31.1 [24.5–38.4]%)	6/183 (3.3 [1.2–7.0]%)	6/57 (10.5 [4.0–21.5]%)
112 days	181	103 (56.9 [49.4–64.2]%)	10/181 (5.5 [2.7–9.9]%)	10/103 (9.7 [4.8–17.1]%)
365 days	135	135 (100.0%)	19/135 (14.1 [8.7–21.1]%)	19/135 (14.1 [8.7–21.1]%)
High burden Group (n = 185)				
14 days	185	70 (37.8 [30.8–45.2]%)	36/185 (19.5 [14.0–25.9]%)	36/70 (51.4 [39.2–63.6]%)
28 days	182	88 (48.4 [40.9–55.9]%)	52/182 (28.6 [22.1–35.7]%)	52/88 (59.1 [48.1–69.5]%)
56 days	176	109 (61.9 [54.3–69.1]%)	71/176 (40.3 [33.0–48.0]%)	71/109 (65.1 [55.4–74.0]%)
112 days	170	126 (74.1 [66.9–80.5]%)	89/170 (52.4 [44.6–60.1]%)	89/126 (70.6 [61.9–78.4]%)
365 days	119	119 (100.0%)	108/119 (90.8 [84.1–95.3]%)	108/119 (90.8 [84.1–95.3]%)

370 patients were divided into risk groups based on CHADS_2_ score (intermediate risk [a CHADS_2_ score of 1] or high risk [a CHADS_2_ score of ≥2]) and AF burden (low AT/AF burden [less than the median average daily AT/AF burden of 0.044 hours/day] or high AT/AF burden [greater than or equal to the median average daily AT/AF burden of 0.044 hours/day]). Data are expressed as means [95% confidence intervals] of estimated AT/AF detection rates with the snapshot monitoring. CIED indicates cardiac implantable electronic device; AT, atrial tachycardia; AF, atrial fibrillation.

The detection rate of snapshot monitoring over various periods (14, 28, 56, 112, and 365 days), calculated based on low/high CHADS_2_ score and low/high AF burden, is shown in Table 3. The detection rate, when used over 365 days of monitoring, was higher for those who had a CHADS_2_ score of ≥2 than for those with a CHADS_2_ score of 1 (53% versus 38%; *P* = 0.03). The detection rate of snapshot monitoring for each time window was higher among the patients with high AT/AF burden than among those with low AT/AF burden (all *P*<0.01). In those with high AT/AF burden, the prolongation of snapshot monitoring up to 365 days increased the detection rate to 91%, while a corresponding value in those with low AT/AF burden was 14%. When we used the cutoff value of 5.5 hours/day in defining high or low AF/AT burden, a threshold associated with a 2-fold increase in thromboembolic events shown in the original TRENDS study [7], results were similar (i.e., the detection rate of 365-day ECG monitoring in those with high AT/AF burden, 86%; a corresponding value in those with low AT/AF burden, 15%).

## Discussion

Using a computer simulation approach in the TRENDS study, we estimated the sensitivity of daily snapshot ECG monitoring over various periods in detecting newly diagnosed AT/AF among the patients with a CHADS_2_ score ≥1 and no history of AT/AF at baseline. The detection rate increased with longer snapshot monitoring, up to 50% when used over 365 days, and shorter intervals of monitoring detected even fewer AT/AF patients. Only 10% of patients with AT/AF were captured by 14 days of snapshot ECG monitoring, and only 15% with 28 days of monitoring. The use of snapshot ECG monitoring to guide clinical care should be implemented cautiously, with the recognition that this method of detection likely underestimates AF episodes.

Reliable and accurate detection of AF is important for decisions on starting or changing anticoagulation or antiarrhythmic therapy in those with stroke risk factors (especially in an aging population). The digital revolution and rapid development of smartphones, mobile connectivity, and social networking have the potential to transform therapeutic approaches [15]. Using a novel smartphone approved by the US Food and Drug Administration [21], patients are currently able to capture and transmit single-lead ECG data to their healthcare providers. Concomitant monitoring of iPhone ECGs and 12-lead ECGs showed that iPhone ECGs had 96–98% sensitivity and 97–98% specificity in recording AF accurately [12,13]. It remains uncertain, however, how often the devices, when used for intermittent monitoring, exactly capture (or miss) paroxysmal AF events. Such evidence is needed before widespread utilization of this new technique.

At least two studies have assessed the sensitivity of intermittent ECG monitoring for detecting AT/AF, comparing it with the findings from concomitant continuous ECG monitoring (i.e., CIEDs). Ziegler et al. suggested that among 547 pacemaker patients with symptomatic AT/AF, the sensitivity (31%-71%) and negative predictive value (21%-39%) were low in several approaches with intermittent monitoring, such as annual, quarterly, or monthly 24-hour Holter ECGs, 7- or 30-day long-term recordings, or symptom-based monitoring [22]. Charitos et al. examined the sensitivity of intermittent ECG monitoring with various durations (i.e., 24 hours, 7 days, 14 days, and 30 days) and frequencies (i.e., 1–12 times) among 647 patients with CIEDs (mean age 69 years) [23]. In both studies [22,23], 80% of the included patients were clinically diagnosed with paroxysmal or persistent AF, and a substantial number of the patients were taking antiarrhythmic drugs at baseline, and thus evidence in more real-world settings is lacking.

An intensification of intermittent ECG monitoring on a number of different days could improve the sensitivity of intermittent ECG monitoring in detecting AF [21,22]. Among patients with stroke risk factors without known AF, 2–4 weeks of intermittent ECG monitoring by a hand-held ECG (<30 seconds of recording twice daily and symptom based-monitoring) could detect AF in 4–7% [14,24]. Whether a longer period of intermittent ECG monitoring (e.g., 365 days), a feasible approach in snapshot ECG monitoring, could improve the sensitivity of intermittent ECG monitoring remains unknown. It also remains uncertain to what extent the intermittent ECG monitoring actually captured (or missed) paroxysmal AF over 2- to 4-week monitoring periods. We found that the daily simulated snapshot ECG monitoring, even if used over 365 days, failed to capture a large proportion of paroxysmal AT/AF. Although our main analyses excluded those with prior histories of AT/AF and those taking warfarin and antiarrhythmic drugs at baseline, 57% of the people have histories of heart failure and 43% have histories of potential sinus node diseases. The incidence and prevalence of paroxysmal AF is higher in CIED users than the population without CIED indication (i.e., general population). Therefore, the current study is not representative of the general population. In addition, we assumed perfect compliance with the daily snapshot monitoring, when in reality this is unlikely (particularly for the longer monitoring windows) [25,26]. The use of smartphone reduces with increasing age, limiting its utility in the elderly, the main target population one may wish to screen for AF. In aggregate, for a general population/clinical practice, the detection rate of snapshot ECG monitoring may be lower than we have found.

The detection rate of snapshot ECG monitoring, when it was performed over 365 days, was higher in those who had a CHADS_2_ score ≥2 points (53%) than in those with a CHADS_2_ score of 1 point (38%). The detection rate also differed by individual AT/AF burden. Only 14% of patients with AT/AF burden <0.044 hours/day were captured, even by 365 days of snapshot ECG monitoring. However, we need to consider that there is still much debate as to whether such a short AF episode actually implies an increased risk of stroke and warrants screening of a population with low AF burden [5,7,27,28]. By contrast, 91% of those with AT/AF burden ≥0.044 hours/day were captured by 365 days of snapshot ECG monitoring. Daily snapshot ECG monitoring may be effective in identifying the high burden patients (i.e., the ones at greatest risk of stroke) if it is used up to 365 days. The results, however, should be interpreted with caution; if one conducts snapshot ECG recordings, as we simulated here, one does not know if the patients identified by this method are those with high or low AT/AF burden. The use of the snapshot is for screening, and then it would be up to the judgment of the ordering physician to decide next steps. For example, more intensive arrhythmia monitoring may be required in those who screen positive in order to provide anticoagulation to those whose amount of AF places them at an increased risk of stroke while avoiding unnecessary anticoagulation (and its attendant bleeding risks) in those whose AF burden places them at low risk of stroke. Further studies are warranted to determine which clinical risk factors identify those who would derive the most clinical benefit from detection of AF by prolonged snapshot ECG monitoring; the cost-effectiveness of this new technology relative to conventional methods of monitoring patients; and the impacts of this technology on the costs of health care via preventing hospitalization for stroke, heart failure, and dementia [15].

This study has some limitations. First, the AT/AF burden data from the device was aggregated on a daily basis, without permitting us to choose specific times within the day and duration of each AT/AF episode to simulate our monitoring or to analyze daytime vs. nighttime onset of AT/AF. Snapshot ECG recording during sleep is not feasible, and thus the detection rate of snapshot ECG monitoring in the current study may be overestimated. Second, the results do not reflect all clinical scenarios since there was no consideration for recordings during symptomatic episodes. However, symptoms believed to be caused by AF have been shown to correlate with the arrhythmia in only 17% of episodes [29].The study is based on a computer model that may not reflect clinical use and may lack direct clinical relevance. Third, the snapshot ECG monitoring used here did not include a concept of a length of measure and how often it performed per day; it shows only the probability of detecting AT/AF in the monitoring window. Fourth, we defined AF as device-detected AT/AF lasting >5 minutes to eliminate atrial oversensing [20]. Numerous studies have supported the cutoff value of >5 minutes AT/AF burden to be associated with thromboembolic event risk [28,30–32]. However, the precise threshold at which thromboembolic event risk increases is still the subject of debate. Fifth, we used the cutoff value of 0.044 hours/day (the median average daily AT/AF burden) as high or low AT/AF burden, which might be arbitrary. To enhance the clinical relevance, we also used the cutoff value of 5.5 hours/day (a threshold associated with thromboembolic event risk [7]) in defining high or low AT/AF burden, and results were similar. Sixth, the comparison of AF/AT detection rates between different window sizes may be subject to bias, since the patients studied for each window size might not be directly comparable. Lastly, intracardiac electrograms were not analyzed, limiting verification of AF diagnosis.

Among the patients with stroke risk factors, daily snapshot ECG monitoring with up to 365 days of follow-up detects AT/AF in half or less than half of patients whose AT/AF is detectable by CIED. The data from the current study raise concerns about the accuracy of daily snapshot ECG monitoring as a tool for detecting AF in those with stroke risk factors. We need to determine the most appropriate applications of snapshot ECG monitoring (e.g., when, how long, for whom), balancing technological possibilities, patient burden, and costs.
